# Paracetamol and Caffeine Combination in Pain Management: A Narrative Review

**DOI:** 10.1155/prm/9166828

**Published:** 2025-07-22

**Authors:** Michel Lanteri-Minet, Rassa Pegahi

**Affiliations:** ^1^Neurology Department, Centre Hospitalier Universitaire de Nice, Nice, Provence-Alpes Côte d'Azur, France; ^2^Medical Department, UPSA SAS, Rueil-Malmaison, Paris, France

**Keywords:** acute pain, antinociceptive effect potentiation, caffeine, combination, pain management, paracetamol

## Abstract

**Background:** Paracetamol is one of the most commonly used analgesic and antipyretic drug, available as a single or a combined formulation. Caffeine is an adjuvant analgesic to several drugs such as paracetamol. The goal of combining paracetamol with caffeine is to achieve a higher analgesic efficacy of paracetamol while lowering its dose and thus reducing side effects.

**Objective:** This narrative literature review aims to provide an overview of the cumulative analgesic effects of this combination and the mechanisms underlying the potentiation by caffeine of the antinociceptive effect of paracetamol.

**Methods:** The search was conducted in PubMed, MEDLINE, ClinicalTrials.gov, and Cochrane Database. For the clinical efficacy and safety, only randomized controlled trials and meta-analysis assessing paracetamol 1000 mg in combination with caffeine 130 mg were considered.

**Results:** As emphasized by the data presented in this review, there is a potentiation of paracetamol-induced analgesia by caffeine with synergistic interactions observed in preclinical and clinical studies. Caffeine enhances the antinociceptive effect of paracetamol and accelerates the absorption of associated paracetamol, which explains the significant faster analgesics' effect with the combination. In clinical trials in patients with mild to moderate acute pain, the combination demonstrates a higher pain relief compared with paracetamol alone with a significant improvement of pain relief in patients with primary headaches without added safety issues.

**Conclusions:** This combination is effective and safe in the treatment of acute mild and moderate pain. Prescribing physicians might consider using paracetamol and caffeine combination among other options in treating these types of pain.

## 1. Introduction

Paracetamol (acetaminophen, N-acetyl-p-aminophenol) has been widely endorsed as a first-line analgesic and is currently the most commonly used analgesic worldwide in a range of pain's types [1]. It has a unique clinical pharmacological profile with a potent analgesic and antipyretic effects [2]. Unlike nonsteroidal anti-inflammatory drugs (NSAIDs), paracetamol does not produce gastrointestinal damage or cardiorenal effects [3], but supratherapeutic dosing of paracetamol may induce severe liver damage [4]. However, at therapeutic doses, paracetamol has a good safety profile, which makes it the recommended analgesic across all ages, especially in elderly and frail patients and in whom NSAIDs are contraindicated [2, 5].

Paracetamol exists as a single chemical entity or in combination with different compounds such as caffeine, NSAIDs, or opioid analgesic drugs. The combination of paracetamol with caffeine is particularly of interest because both components have a distinct mechanism of action (MoA) [1, 5, 6]. While the mechanism of the potentiation of paracetamol-induced analgesia by caffeine remains a subject of discussion, preclinical and clinical studies provided substantial evidence in support of the caffeine contribution to improve the effectiveness of paracetamol. The combination of paracetamol and caffeine has been the topic of several studies over the last decades, and it has been evaluated in different categories of pain with different doses of paracetamol and caffeine. The recommended initial dose in the treatment of mild to moderate pain and/or fever in adults and adolescents aged upper 15 years is a tablet of paracetamol 500 mg combined with 65 mg of caffeine with a maximum single dose of 1000 mg of paracetamol and 130 mg of caffeine (2 tablets) [7]. A preliminary review of literature revealed that the combination of 1000 mg of paracetamol with 130 mg of caffeine was the most assessed dosage in randomized clinical trials regardless of pain's type. According to a meta-analysis conducted in acute pain, the addition of caffeine at doses ≥ 100 mg to analgesics provides an increase in the proportion of participants who experience a good level of pain relief [8]. Similarly, Renner et al. in a pharmacokinetic–pharmacodynamic study reported that caffeine at a dose of 130 mg added to 1000 mg of paracetamol causes a significantly enhanced and sustained antinociceptive effect of paracetamol [9]. For all the above, we decided to restrain our research to the combination of 1000 mg of paracetamol with 130 mg of caffeine. Therefore, the purpose of this review paper is to review the efficacy and safety data of the combination of paracetamol 1000 mg with caffeine 130 mg in treating different types of pain and to discuss the knowledge regarding the cumulative analgesic effects in one hand and the synergistic analgesic effects of this combination.

## 2. Methods

This literature review includes studies on the association of paracetamol and caffeine (preclinical studies and clinical trials in different types of pain). Clinical databases including PubMed, MEDLINE, ClinicalTrials.gov, and Cochrane Database of Systematic Reviews were searched from inception to March 2024. The following combinations of keywords were used to search the potential literature: “acetaminophen/paracetamol and caffeine combination/association” and “pharmacokinetics of acetaminophen/paracetamol and caffeine.” Other keywords were also used: “mechanisms of action of acetaminophen/paracetamol and caffeine,” “acetaminophen/paracetamol review,” “caffeine review,” and “caffeine and pain.” The inclusion criteria were as follows: English-language publications, randomized double blinded clinical trials, meta-analysis, and systematic review evaluating “the combination of 1000 mg of paracetamol with 130 mg of caffeine versus paracetamol 1000 mg alone or any other analgesic,” pharmacokinetics studies evaluating the combination of paracetamol with caffeine, and literature review on the MoA of paracetamol and caffeine. Studies evaluating either other dosing of paracetamol and caffeine combination or other paracetamol's combination were excluded. So, for eligibility criteria, randomized controlled trials exploring the combination of paracetamol 1000 mg with caffeine 130 mg in comparison with paracetamol alone 1000 mg in any type of pain were considered for inclusion in the review.

In the following, we will describe the main pharmacological features of paracetamol and caffeine and will review preclinical and clinical studies that have investigated the combination of both compounds in several types of pain.

## 3. Paracetamol Metabolism and Mode of Action

### 3.1. The Metabolism of Paracetamol

Paracetamol has a high oral bioavailability (88%), it is well absorbed with peak plasma concentrations occurring about 30 min–2 h after ingestion [7]. Paracetamol is not widely bound to plasma proteins; it has a plasma half-life of 1.5–2.5 h at the recommended doses [10]. At therapeutic dose, glucuronidation (50%–70%) is the main pathway of paracetamol metabolism, followed by sulfation (25%–35%) and a minor fraction (5%–15%) is oxidized by cytochrome P450 2E1 (CYP2E1) to generate N-acetyl-p-benzoquinoneimine (NAPQI). NAPQI is a reactive intermediate normally rapidly neutralized by conjugation with glutathione, which is constantly replenished. Very high doses of paracetamol result in formation of excess NAPQI with the depletion of GSH levels causing mitochondrial dysfunction and liver necrosis [10]. About 3% of the drug is excreted unchanged in the urine [11]. Paracetamol crosses the blood–brain barrier with ease and is distributed homogeneously throughout the central nervous system (CNS) [12].

### 3.2. The Mechanism of Analgesia of Paracetamol

Although paracetamol has been widely used in clinical practice for more than a century, its exact MoA remains debated. While the antipyretic activity of paracetamol is explained by the inhibition of prostaglandin (PG) synthetase in the brain, the precise analgesic action of paracetamol is still under investigation and seemingly not solely mediated through cyclooxygenase 1-2 (COX) inhibition [1, 3, 13]. The inhibition of a third COX isoenzyme (COX 3) by paracetamol has been explored; however, while some authors consider it as a potential target of paracetamol's action, others refute this hypothesis [1, 4, 14]. The data produced by more recent studies show that the mechanisms responsible for paracetamol's antinociceptive effect are located in the CNS and are interlinked [3, 15]; besides, we now know that paracetamol is converted mainly in the brain to its active metabolite AM404 (N-arachidonoylphenolamine) in a multistep process [16,17]. AM404 is a potent activator of TRPV1, a major contributor to neuronal response to pain. Activated TRPV1 at the peripheral level induces nociception, hyperalgesia, and pain transduction modulation. On the contrary, supraspinal TRPV1 activation induces antinociception [18, 19]. Several studies have provided evidence on the involvement of serotoninergic pathway in the analgesic effect of paracetamol, the selective blockade of serotoninergic receptors has been shown to abolish the analgesic actions of paracetamol in acute models of pain [20]. The blockade of cannabinoid CB1 receptors with a CB1 receptor antagonist or in CB1 receptor knockout mice prevents the analgesic action of paracetamol in animal models [3, 17].

Thus, paracetamol has a distinct and multidimensional analgesic MoA where AM404 plays a key role in several pain pathways within the CNS such as endocannabinoid, serotoninergic, and nitric oxide. All these pathways are involved in acute and chronic pain states [1, 21].

## 4. Caffeine Metabolism and Mode of Action

### 4.1. The Metabolism of Caffeine

Caffeine also known as trimethylxanthine is a chemical compound found in coffee. Caffeine has a rapid absorption at intestinal level after oral administration and reaches 99% in human blood in about 45 min after ingestion [22]. The oral absorption of caffeine is complete and fast with peak plasma concentrations observed approximately 30–60 mm after oral consumption. Caffeine displays limited protein binding (10%–35%). The plasma half-life of caffeine in humans is generally 3–5 h, and the kinetics of caffeine have been reported to be linear in humans [23] with less than 2% of a caffeine dose eliminated unchanged in urine [22]. Caffeine crosses both the blood–brain and placental barriers and it is secreted in human milk, saliva, bile, and semen [23]. About 95% of a caffeine dose is metabolized in the liver via cytochrome P450 (CYP1A2 demethylation), which converts it into paraxanthine (85%), theobromine (10%), and theophylline (5%) [22, 24].

### 4.2. The MoA of Caffeine as an Analgesic and Adjuvant Analgesic

Caffeine may exert an antinociceptive effect in the brain. This effect is primarily attributed to its nonselective antagonism of the adenosine A1, A2A, A2B, and A3 receptors (as caffeine and adenosine receptors possess a similar molecular structure) [25, 26]. Caffeine antagonizes all human adenosine receptors with similar affinity [25]. While several studies have observed an antinociceptive effect of caffeine in some animal models of pain, other studies have failed to show any analgesic effect, so a direct analgesic effect of caffeine when given alone is still a matter of debate [23, 27]. Differences in experimental conditions and in pain intensities might explain these conflicting results [27]. Even though caffeine has been reported to be effective against certain states of human pain, such as headache, we do not know whether the type and the pathophysiology of pain can explain some of the conflicting results of caffeine effect [27, 28].

Caffeine is generally not considered as an inhibitor of PG synthesis; however, Fiebich et al. reported that caffeine like paracetamol inhibited PGE2 synthesis in lipopolysaccharide (LPS)-stimulated rat microglial cells. Furthermore, caffeine and paracetamol showed synergistic activity in the same system since the combination of paracetamol with 30 μM of caffeine was about 7 times more potent than paracetamol alone in inhibiting LPS-induced PGE2 synthesis and that caffeine significantly inhibits COX-2 protein synthesis. The authors suggested that the inhibition of COX in microglial cells contributes to the adjuvant analgesic activity of caffeine [29]. The same authors in another study found that COX-2 is induced in microglial cells by the activation of adenosine A2A receptors [30]. In a more recent study in healthy subjects, Renner et al. observed synergistic effects of caffeine and paracetamol; the authors advanced the hypothesis that caffeine might enhance the antinociception of paracetamol through the activation of inhibitory glycinergic transmission (the blockade of prostaglandin E2 (PGE2) production curbs glycinergic inhibition of nociception in the spinal cord postsynaptically) [9].

## 5. Preclinical and Clinical Data of Paracetamol and Caffeine Combination

Caffeine is present in several analgesic preparations, and it is currently used as an adjuvant. The effects of paracetamol in combination with caffeine have been evaluated in preclinical and clinical studies and in different types of pain.

### 5.1. Preclinical Data

In experimental studies in rats, caffeine has been shown to increase the antinociceptive effects of paracetamol. However, this potentiation occurs only if adequate dose combinations are used. Siegers evaluated 9 paracetamol and caffeine dose combinations (100, 200, and 400 mg/kg of paracetamol and 10, 50, and 100 mg/kg of caffeine). The authors observed that caffeine depresses paracetamol serum levels in a dose-dependent way with a significant decrease of paracetamol serum concentration with 10 mg/kg of caffeine; this was associated with a significant decrease of the paracetamol-induced analgesia between the 30th and the 60th min, while at doses 50 mg/kg and 100 mg/kg, caffeine increases paracetamol analgesia [31].

Granados-Soto et al. examined 16 paracetamol and caffeine dose combinations and a maximal potentiation of paracetamol analgesic effect was observed with 316 and 32 mg/kg of paracetamol and caffeine. This combination of 316–32 mg/kg paracetamol–caffeine did not modify paracetamol plasma level compared with paracetamol 316 mg/kg alone [32].

The coadministration of caffeine with other analgesics showed that caffeine increases the antinociceptive response of analgesics at some but not at all doses [27, 33].

The inhibition of paracetamol's antinociception by caffeine at 10 mg/kg has been examined by two animal studies, and this inhibition seems to be related to the blockade of adenosine A1 receptors which mediate antinociceptive actions [6, 34].

### 5.2. Clinical Data (Pharmacokinetic) in Healthy Subjects

The influence of caffeine on the pharmacokinetic characteristics of paracetamol has been assessed in some healthy subject's studies with different doses of paracetamol and caffeine. Renner et al. showed in a PK–PD study that caffeine accelerates the absorption of associated paracetamol as indicated by increased early AUCs with a significant faster analgesics' effect of the combination on tonic pain ratings as compared with paracetamol alone [9]. In another study, Iqbal et al. demonstrated that caffeine induces an increase in paracetamol bioavailability and absorption rate (significant increase of AUC and Cmax of paracetamol compared with paracetamol alone) and a decrease of paracetamol clearance [35]. A faster absorption for the combination compared with paracetamol alone has also been showed by Guzman et al. [36]. These findings may be attributed to an enhanced blood flow in the gastrointestinal mucosa with a possible decrease in early clearance [35, 37]. According to Iqbal et al., the slower clearance is not likely to add to the toxicity of paracetamol at the dose studied since the mechanism purportedly responsible for paracetamol toxicity is not likely to be affected and, in the light of the recent reports, caffeine may even act as hepatoprotective, reducing the toxicity of paracetamol [35].

A number of randomized controlled trials have assessed the analgesic efficacy of the combination of P1000 mg–C130 mg in primary headaches/migraine, in postoperative and postpartum pain, in pain of dysmenorrhea, and in dental pain. Characteristics and main data of clinical trials reviewed in this paper are summarized in Table 1.

### 5.3. The Efficacy and Safety of Paracetamol and Caffeine Combination in Primary Headaches

Headache is one of the most prevalent disorders worldwide [43]. Migraine is an intermittent neurological disorder that affects 10%–12% of the western population [41]. Tension-type headache (TTH) is a more common condition with a worldwide prevalence of at least 40% [44]. The majority of patients (59%) reported TTH one day each month or less and 24%–37% had headaches several times each month [45]. Migraine and TTH are both associated with a significant burden on individual and society levels [24].

#### 5.3.1. The Efficacy and Safety of Paracetamol and Caffeine Combination in TTH

The combination of P1000 mg–C130 mg has been compared with P1000 mg alone in TTH patients through two randomized double blinded clinical studies (Study 1 and Study 2) carried out by Migliardi et al. [39].

In a study, 1, 336 patients were treated with the combination P1000 mg–C130 mg and 332 patients with P1000 mg alone. In another study, 2, 339 patients were treated with the combination P1000 mg–C 130 mg and 337 patients with P1000 mg alone.

Demographic and headache history of included patients showed a mostly moderate to severe headache. The mean number of headache days reported per month in Study 1 and Study 2 were 30 and 10, respectively. Two periods of treatment were proposed, each period with two headache attacks. At least 48 h had to elapse between treatment of the first and second headache attack. A 7-day washout at least separated the Period 1 and Period 2. During the washout period, patients took their own usual analgesic if needed. Patients had to abstain from caffeine-containing products for the 4 h following self-administration of the study drugs. Pain intensity was evaluated using the sum of pain intensity differences (SPIDs), the percentage of SPID (% SPID), and total pain relief (TOTPAR) scores. In both studies, the combination P1000 mg–C130 mg was significantly superior to P1000 mg alone (*p* < 0.01) for SPID, %SPID, and TOTPAR. For the pooled data, the increment in analgesic effect achieved by combining P1000 mg–C130 mg was 83%, 76%, and 89% of the net paracetamol effect for SPID, %SPID, and TOTPAR, respectively. The influence of caffeine consumption on the analgesic effect of tested drugs has also been evaluated in this study. The mean SPID scores were significantly higher in the combination versus paracetamol alone and independently of caffeine consumption (*p*=0.019 for users and *p*=0.002 for nonusers of caffeine); this would suggest that the adjuvant effect of caffeine is not attributed to relieving caffeine withdrawal headache. A greater incidence of nervousness and dizziness was reported in the P1000 mg–C130 mg group (7% and 5%) compared with P1000 mg alone group (1% and 2%), respectively. Any side effect was reported in 21% in the combination group versus 13% in the paracetamol group [39].

The aim of the clinical trial led by Pini et al. was to confirm in an Italian population affected by TTH the good safety and tolerability profile of the combination P1000 mg–C130 mg observed in previous studies and to compare this combination with naproxen sodium 550 mg. The efficacy of these two active treatments was a secondary objective. Ninety-nine patients were included in this randomized double blind and crossover study. The majority of patients (98%) reported a mean monthly number of TTH days between 4 and 14 with moderate intensity in 75.8% of the patients. The daily consumption of coffee was at least two cups of coffee. The tolerability assessments were available in 93 patients. The difference between treatments was nonsignificant. The adverse events with a frequency ≥ 10% recorded in 4-h period after ingestion of P1000 mg–C130 mg or naproxen 550 mg were nervousness (14.5% and 12.1%), nausea (19.7% and 22.7%), drowsiness (14.5% and 21.2%), fatigue (15.8% and 15.2%), and dyspepsia (7.9% and 10.6%), respectively. Dizziness was only reported with the combination P1000 mg–C130 mg. To assess the efficacy, pain intensity and pain relief were evaluated hourly during the 4-h post dose period of each treatment, SPID, and TOTPAR were calculated. Both treatments were not significantly different from each other [41]. Yue et al. compared the combination of P1000 mg–C130 mg to another NSAID, ibuprofen 400 mg in patients with TTH (rapid acting paracetamol formulation). The authors reported a comparable efficacy between the combination of P1000 mg–C130 mg and ibuprofen 400 mg [46].

#### 5.3.2. The Efficacy and Safety of Paracetamol and Caffeine Combination in Migraine

The combination of P1000 mg–C130 mg was evaluated in migraine attacks in one clinical trial. This combination was compared with sumatriptan (SUM) 50 mg as an active control. The primary objective of the study was to show the efficacy of the combination of P1000 mg–C130 mg in reducing pain in migraine attacks and its tolerability in migraine treatment. One hundred and eight patients participated to this randomized, double-dummy, cross-over, drug-controlled trial. All patients had the diagnosis of migraine (ICHD-II criteria) with or without aura with a mean frequency of 2–8 attacks per month. The daily consumption of coffee was at least two cups of coffee. Pain intensity and pain relief were evaluated hourly during the 4 h post dose period of each treatment, and SPID and TOTPAR were calculated. The data showed that both treatments were effective with no difference between them. There was no safety difference between the treatments except a slight increase of referred fatigue in patients assuming SUM. The intensity of side effects was always slight or moderate, all side effects disappeared spontaneously, and none requested any modification of scheduled treatment [42].

In conclusion, data presented above confirm that the addition of 130 mg of caffeine to 1000 mg of paracetamol improved significantly pain relief versus paracetamol alone in TTH patients. Similarly, in patients with migraine, this combination showed a comparable efficacy and safety to SUM. Paracetamol and caffeine combination is safe and effective in treating TTH and migraine attacks. Prescribing physicians might consider the option to use paracetamol, especially combined with caffeine in early treatment of acute migraine pain among other more costly or less safe options [47]. However, as observed with some drugs such as combination analgesics, opioids or triptans, there is a risk of developing medication overuse headache [48, 49] and caffeine-withdrawal headache. Guidelines from the German, Austrian, and Swiss Headache Societies and the German Society of Neurology recommend paracetamol as a first-line option for self-medication of migraine attacks and the fixed-dose combination of paracetamol, aspirin, and caffeine and the combination of paracetamol and caffeine as first-line therapies for TTH. The recommendation states, however, that headache and migraine medications should not be taken for more than three consecutive days and on more than 10 days per month [50]. According to Barbanti et al. and the Italian experts' panel, the use of the combination of paracetamol and caffeine as first-line treatment of migraine does not limit in any way further therapeutic options provided that patients were informed and educated about the possible overdosage of caffeine [47].

### 5.4. The Efficacy and Safety of Paracetamol and Caffeine Combination in Postpartum Pain and Dysmenorrhea Pain

Postpartum pain includes pain due to episiotomy and uterine cramp after childbirth, and pain intensity is generally moderate to severe [28].

Laska et al. assessed the combination of paracetamol and caffeine in 3 randomized controlled studies versus paracetamol alone versus placebo with different doses in postpartum pain. Only the data relating to the combination P1000 mg–C130 mg in comparison with P1000 mg will be described.

Study 1 and Study 2 included subjects with postepisiotomy/postsurgical pain and uterine cramping whereas Study 3 was limited to postepisiotomy/postsurgical pain. The authors included in the 3 studies a total of 396 patients randomly assigned to single doses of either P1000 mg–C130 mg (*n* = 197) or P1000 mg alone (*n* = 199). Pain intensity was measured at the time medication, 30 min after medication and hourly thereafter for 4 h. The SPID, % SPID, TOTPAR, and time to onset of pain relief (ONSET) were measured. Mean values for %SPID, TOTALPAR, SPID, and ONSET indicated superiority for the combination treatment over paracetamol alone in the three studies with a significant difference in Study 1. When stratified according to the pain etiology, the average response for all treatments is persistently higher in the group with uterine cramping than in the episiotomy/surgery pain group; this observation confirmed data reported by Laska and Sunshine et al. in earlier research. In addition, the pooled relative potency based on %SPID and stratified on pain etiology in the presence of caffeine was 2.3 (95% confidence interval (CI) not finite) in the uterine cramping group versus 1.6 (95% CI: 1.1–2.9) in the episiotomy/surgery pain group [38]. This would suggest that caffeine has a higher adjuvant effect in the presence of the more intense stimulus [23]. As regards to safety, the combination has been well tolerated with no reported adverse events.

Primary dysmenorrhea is a highly prevalent condition of premenstrual women (50% of menstruating females) and causes significant disruption in quality of life and absenteeism. Dysmenorrhea is related to uterine hypercontractility, which induces pain [51]. An intense secretion of PG would mediate uterine contraction, and NSAIDS such as ibuprofen and naproxen are efficacious and commonly used as treatment for primary dysmenorrhea. However, other mediators have been implicated and this might explain partial or no relief from NSAIDs observed in some women [52]. Ali et al. evaluated in 320 women with moderate to severe dysmenorrhea pain the combination of P1000 mg–C130 mg in comparison with P1000 alone. In this study, subjects assessed in a diary are as follows: period pain, period pain relief, cramping, backpain, and tiredness levels at each time point. The primary efficacy measure of TOTPAR over 0–2 h was met with a significant difference in favor of the combination at 30 min after dosing (*p* < 0.05), and this difference was maintained for the duration of the study (6 h). Significant differences in favor of P1000 mg–C130 mg combination were also observed in secondary efficacy parameters evaluating the cramping and the backpain intensity scores and tiredness (*p* < 0.05). No serious adverse events were reported in the study, and two adverse events were causally related to P1000 mg–C130 mg combination while one to P1000 mg alone [40].

In conclusion, there is evidence of additional efficacy of caffeine when combined with paracetamol in postpartum pain (three studies) and in dysmenorrhea pain (one study).

### 5.5. The Efficacy and Safety of Paracetamol and Caffeine Combination in Dental/Surgery Pain

Dental impaction pain model (DIPM) is frequently used to evaluate analgesic efficacy as it's easily adapted to perform multiple-dose studies, pharmacokinetics/pharmacodynamics correlations, preemptive interventions, and sleep–pain studies [53]. Main analgesic agents were evaluated in DIPM including paracetamol, NSAIDs, opioid agents, and combination therapy [54].

Laska et al. compared in dental extraction pain, the combination of P1000 mg–C130 mg (*n* = 45) with P1000 mg alone (*n* = 46). The mean response (SPID, %SPID, and TOTAL) to P1000 mg–C130 mg was higher than P1000 mg alone but not statistically significant with no reported adverse events. The authors quoted that while it may be concluded that the combination of paracetamol and caffeine is superior to paracetamol alone, the amount of paracetamol required for the same effect as a given dose of the combination cannot be estimated from the data in this study [38]. In another study on dental pain, Winter et al. showed no difference of the combination of paracetamol at 1000 mg with caffeine at 135 mg versus paracetamol 1000 mg alone but of the patients constituting the combination group, 5 of 40 had severe pain, whereas in the paracetamol group alone, 13 of 41 had severe pain [55].

Others clinical trials have evaluated the combination of paracetamol with caffeine but at lower doses than P1000 mg–C130 mg in comparison with several active treatments in different dental pains.

We cannot draw any conclusion on a higher efficacy of P1000 mg–C130 mg over 1000 mg alone in dental pain as the trials evaluated different doses and different types of dental pain.

### 5.6. Paracetamol and Caffeine Combination in Meta-Analysis

The meta-analysis published by Zhang and Po assessed the efficacy and safety of the combination of paracetamol with caffeine and the combination of paracetamol with codeine at several doses and investigated in different range of pain types. The authors conclude that the combination of paracetamol and caffeine was superior, but the effects were weak [56]. Palmer et al. undertook a more recent meta-analysis with the assessment of the benefit of adding 130 mg of caffeine to 1000 mg of paracetamol in the treatment of mild to moderate pain. Eight studies across a number of acute pain states (dysmenorrhea, headache, postpartum pain, and dental pain) were included in the review. The authors conclude that the relative benefit (of achieving at least 50% pain relief) of paracetamol–caffeine vs paracetamol alone was 1.12 (95% CI: 1.05–1.19, *p*=0.0004) [57].

### 5.7. Interactions of Paracetamol and Caffeine and Clinical/Safety Implications

Our review provides evidence that in animal studies, caffeine at certain doses and in certain pain states potentiates paracetamol-induced antinociception [6, 9, 31–34], and in clinical trials, the addition of 130 mg of caffeine to 1000 mg paracetamol increases the analgesic effect of paracetamol in different types of pain [38–42, 46, 56, 57]. In TTH/migraine, adding caffeine to paracetamol increased significantly the number of patients who experienced pain relief in comparison with paracetamol alone. The efficacy observed with this combination is potentially due to a centrally located analgesic action of both compounds. Caffeine may relieve headache symptoms by increasing cerebrovascular tone and decreasing cerebrovascular volume and flow (secondary to adenosine receptor blockade) [58]. In addition to this effect, caffeine may act as an analgesic given its ability to inhibit the synthesis of leukotrienes and PGs, which are clearly involved in the pathophysiology of migraine [59]. Paracetamol is highly effective in relieving the pain of migraine and TTHs [2, 60]; this could be related to its potent inhibition of PGE2 in the brain [20]. Caffeine has been shown to inhibit PGE2 synthesis in rat microglial cells via the blockade of COX-2 protein synthesis [29]. Thus, the inhibition of COX might contribute to the adjuvant analgesic activity of caffeine observed in TTH/migraine clinical trials. Transient receptor potential (TRP) channels are found to have high levels of expression in the CNS with involvement in a wide range of pathologies, including migraine headache, epilepsy, pain, and inflammation [61]. The activation of transient receptor potential vanilloid-1 (TRPV1) in the brain by the bioactive metabolite of paracetamol AM404 contributes to the analgesic effect of paracetamol [19]. Caffeine is also suggested to be an activator of TRPV1, and this could explain a potential mechanism for caffeine's adjuvant analgesic [61]. So far, there are no published data assessing the effect of the double activation of TRPV1 by paracetamol and caffeine on the antinociceptive effect of paracetamol.

We notice an additional efficacy of caffeine when combined with paracetamol in treating postpartum pain (postepisiotomy/postsurgical pain or uterine cramping) with higher average response for the combination in the uterine cramping than in the postepisiotomy/postsurgical group [38]. As for dental extraction pain, the analgesic adjuvant effects of caffeine are inconclusive [38]. Weak adjuvant effects of caffeine in patients with postoperative pain have been observed with the combination of caffeine with acetylsalicylic acid or ibuprofen [24]. The differences observed with the analgesic adjuvant effects of caffeine would be related to the differences in the types of pain and the intensity of pain.

In this clinical review, we observe no evidence for a higher prevalence of adverse events of the combination P1000 mg–C130 mg in comparison to P1000 mg alone, and no serious or unexpected adverse events have been reported in these trials.

The safety profile of paracetamol and caffeine is well documented, and we know that high dose of paracetamol can lead to acute hepatic necrosis. Whether caffeine exacerbates or protects against hepatotoxicity has been the subject of some debate. Animal and in vitro studies are controversial; some studies concluded that caffeine has a stimulatory effect on paracetamol-induced hepatoxicity in rats via the enhancement of NAPQI production and the increase of formation by CYP3A4 of paracetamol–glutathione conjugates [62–64]. This stimulatory effect seems to be exerted directly; however, the exact mechanism remains to be elucidated [65]. On the other hand, other studies showed an hepatoprotective role of caffeine against paracetamol-induced liver toxicity in mice by prevention of reduced glutathione depletion [66, 67]. This inhibitory effect of caffeine on paracetamol oxidation by microsomes may be suggested by the potential antioxidant role for caffeine. Gonçalves et al. evaluated parameters associated with mitochondrial function and oxidative stress on livers of mice treated with caffeine and paracetamol. The authors found that caffeine does not increase the hepatoxicity induced by paracetamol but rather improved hepatic mitochondrial respiration, possibly through its antioxidant properties and/or via its interactions with mitochondrial purinergic receptors (prevention of lipid peroxidation and the ROS production in mice livers) [68]. The molecular mechanism by which caffeine and antioxidants reduce liver damage would be due to their ability to maintain liver cell integrity [69].

The discrepancies observed between these studies may be explained by the difference in induction state of the liver and the difference in the relative affinities of paracetamol and caffeine for hepatic P450s in mouse and rat [63, 66]. Palmer et al. performed an interesting analysis of this interaction in their meta-analysis and concluded that there are no compelling data to suggest a clinically meaningful increase in hepatotoxicity with the use of paracetamol/caffeine combination [57]. Unfortunately, there are no data in human and transposability of animal and in vitro data to humans is challenging knowing that in these studies, paracetamol and caffeine were used at supratherapeutic doses. In conclusion, the question of the inhibitory and/or stimulatory effects of caffeine on the hepatic metabolism of the paracetamol remains open and requires more in vitro studies.

With regard to caffeine safety, the most reported adverse events and attributable to caffeine observed in this review were nervousness and dizziness. None of the patients in these studies reported any withdrawal symptoms or dependence. However, chronic repetitive exposures to caffeine increase the risk of a dependence syndrome and sudden cessation of caffeine use after chronic exposures (> 200 mg/day for more than 2 weeks (International Classification of Headache Disorders (ICHD-3) [70]) leads to a withdrawal syndrome with headache as a dominant symptom [24]. The withdrawal syndrome would be explained by overregulation and hypersensitivity of adenosine receptors. With the abrupt cessation of caffeine consumption, adenosine receptors become available, leading to vasodilation and significant increase in cerebral blood flow [71]. The potential for physical and psychological dependence on caffeine-containing analgesics has been investigated and did not show any more patients with headaches among those treated with caffeine-containing analgesics compared with those with analgesic alone [56].

## 6. Paracetamol and Caffeine Combination in Special Populations

Efficacy of the combination of paracetamol and caffeine has not been evaluated in special populations but some pharmacokinetic data are available to better control safety in special populations.

El-Lakkany et al. investigated the involvement of liver dysfunction in the modulation of paracetamol 1000 mg pharmacokinetic profile (PK) when combined with caffeine 130 mg versus paracetamol alone 1000 mg in healthy volunteers and in a group of HCV cirrhotic patients (child-Pugh B). Healthy subjects exhibited an increase in Cmax with a decrease in Tmax of paracetamol when compared with subjects treated with paracetamol alone. In cirrhotic patients, as expected, liver impairment modified the PK of paracetamol with a decrease in its clearance; caffeine induced a faster absorption (shorter Tmax and higher Ka) and prolonged t1/2 of paracetamol [72].

Paracetamol is the analgesic of choice in elderly patients [54, 73] and rates of caffeine clearance are similar in older and younger adults [23]. It seems reasonable to consider the careful use of the combination of paracetamol with caffeine effective and safe in elderly patients with migraine [47]. However, elderly patients may require dosage adjustments for certain medications as they have age-related slower metabolisms and/or age-related renal impairment.

In pregnant and breastfeeding women, a prolongation of half-life of caffeine (2.5–7 times) occurs during late pregnancy and caffeine crosses the placental barriers [23]. So, the combination of paracetamol and caffeine is not recommended in treating pregnant and breastfeeding women.

## 7. Review Limitations

This narrative review has some potential limitations as the data presented in the review do not represent all the studies evaluating the combination of paracetamol and caffeine and the search strategy was limited to the English language. Besides, no systematic review of literature was performed and, therefore, some relevant studies may have been missed. In this narrative review, we did not proceed to any comparison with any other caffeine-containing analgesic combinations. Similarly, we could not make any comparison of the efficacy and safety profile of the combination according to the type of pain. Nevertheless, all the presented studies assessing the combination of P1000 mg–C130 mg in this review are randomized double blinded trials with a significant number of patients included in different types of pain.

## 8. Conclusion

Our paper is one of few literature reviews on the efficacy and the safety of the combination of paracetamol 1000 mg with caffeine 130 mg in different pain types. There is a clear potentiation of paracetamol-induced analgesia by caffeine as emphasized by the data presented in this review. In fact, in TTH and migraine, dental pain, postpartum pain, and dysmenorrhea clinical trials, adding caffeine at a dose of 130 mg–1000 mg of paracetamol results in a greater analgesic effect than is achieved with paracetamol 1000 mg alone. The magnitude of caffeine adjuvancy in TTH studies seems to be greater than that which would be predicted from the studies of postpartum pain and other type of pain. Paracetamol 1000 mg and caffeine 130 mg combination shows a favorable efficacy and safety profile. Such combination deserves to be considered by guidelines as a therapeutic option in the management of mild to moderate pain. To further consolidate the good safety profile of this combination, practitioners are encouraged to include their patients in real life cohorts.

## Figures and Tables

**Table 1:** Clinical trials assessing the combination of paracetamol 1000 mg with caffeine 130 mg in different types of pain.

Study	Design	Pain state	Comparisons	No. of participants	Efficacy	Safety
Laska et al. [38] (3 studies)	Double blind parallel groups	Postpartum	P (1000 mg)–C (130 mg) vs. P (1000 mg)	197 (P–C)	SPID, %SPID, TOTPAR and ONSET: P–C vs. P; *p* < 0.05	Vomiting reported in one patient (P) and was dropped from the study
				199 (P)		
Laska et al. [38]	Double blind parallel groups	Dental surgery	P (1000 mg)–C (130 mg) vs. P (1000 mg)	45 (P–C)	SPID, %SPID, TOTPAR and ONSET: P–C vs. P; *p* < 0.05	No reported AE
				46 (P)		
Migliardi et al. [39] (2 studies)	Double blind crossover	Episodic tension headache	P (1000 mg)–C (130 mg) vs. P (1000 mg)	675 (P–C)	SPID, %SPID, TOTPAR: P–C vs. P; *p* < 0.05	AE reported (in P–C vs. P):
				669 (P)		Any: 21% vs. 13%
						Stomach discomfort: 9% vs. 7%
						Nervousness: 7% vs. 1%
						Dizziness: 5% vs. 2%
Ali et al. [40]	Double blind crossover	Primary dysmenorrhea	P (1000 mg)–C (130 mg) vs. P (1000 mg)	298 (P–C)	Primary endpoint: TOTPAR_0–2 h_: P–C vs. P; *p* < 0.05. P–C	14.4% reported at least one AE: 5% (P–C) vs. 5.6% (P)
				301 (P)	TOTPAR_0–3 h,_ TOTPAR_0–4 h_, TOTPAR_0–6 h_, SACID and SBID, P–C vs. P; *p* < 0.05	The three most common AE were headache, nausea, and dizziness
Pini et al. [41]	Double blind crossover	Episodic tension headache	P (1000 mg)–C (130 mg) vs. NAP (550 mg)	91 (in the efficacy evaluation)	Both treatments are effective	Reported AE ≥ 10% (in P–C vs. NAP):
					PID, SPID, TOTPAR: ns P–C vs. NAP	Nervousness: 14.5% vs. 12%
						Nausea: 19.7% vs. 22.7%
						Drowsiness: 14.5% vs. 21.2%
						Fatigue: 15.8% vs. 15.2%
						Dyspepsia: 7.9% vs. 10.6%
Pini et al. [42]	Double blind crossover	Migraine attack	P (1000 mg)–C (130 mg) vs. SUM (50 mg)	92	Both treatments are effective	Reported AE ≥ 10% (in P–C vs. SUM):
					SPID, TOTPAR: ns P–C vs. SUM	Nervousness: 10% vs. 14.3%
						Palpitations: 9.1% vs. 11.6%
						Nausea: 28.2% vs. 22.3%
						Drowsiness: 14.5% vs. 21.2%
						Fatigue: 13.6% vs. 17.9%
						Dyspepsia: 5.5% vs. 15.1%

*Note:* C: caffeine; NAP: naproxen; P: paracetamol; SUM: sumatriptan.

Abbreviations: AE = adverse event, ns = nonsignificant, SACID = sum of abdominal cramping intensity differences, SBID = sum of backache intensity differences, SPID = sum of pain intensity differences, TOTPAR = total pain relief.

## Data Availability

The data that support the findings of this study are available from the corresponding author upon reasonable request.
